# miR-524-5p of the primate-specific C19MC miRNA cluster targets TP53IPN1- and EMT-associated genes to regulate cellular reprogramming

**DOI:** 10.1186/s13287-017-0666-3

**Published:** 2017-09-29

**Authors:** Phan Nguyen Nhi Nguyen, Kong Bung Choo, Chiu-Jung Huang, Shigeki Sugii, Soon Keng Cheong, Tunku Kamarul

**Affiliations:** 1 Centre for Stem Cell Research, Universiti Tunku Abdul Rahman, Sungai Long, Kajang, Selangor DE Malaysia; 2 Department of Preclinical Sciences, Faculty of Medicine and Health Sciences, Universiti Tunku Abdul Rahman, Sungai Long Campus, Bandar Sungai Long, Cheras, 43000 Kajang, Selangor DE Malaysia; 3 Faculty of Medicine and Health Sciences, Universiti Tunku Abdul Rahman, Sungai Long, Kajang, Selangor DE Malaysia; 4 Department of Animal Science, Chinese Culture University, Taipei, Taiwan; 5 Graduate Institute of Biotechnology, Chinese Culture University, Taipei, Taiwan; 6 Singapore BioImaging Consortium A*Star, Singapore, Singapore; 7 Duke-NUS Graduate Medical School, Singapore, Singapore; 8 Tissue Engineering Group, National Orthopaedic Centre of Excellence for Research and Learning, Kuala Lumpur, Malaysia; 9 Department of Orthopaedic Surgery, Faculty of Medicine, University of Malaya, Kuala Lumpur, Malaysia

**Keywords:** C19MC, miR-524-5p, Reprogramming efficiency, Cell proliferation, Apoptosis, Pluripotency genes, MET, TP53INP1, ZEB2, SMAD4

## Abstract

**Background:**

Introduction of the transcription factors Oct4, Sox2, Klf4, and c-Myc (OSKM) is able to ‘reprogram’ somatic cells to become induced pluripotent stem cells (iPSCs). Several microRNAs (miRNAs) are known to enhance reprogramming efficiency when co-expressed with the OSKM factors. The primate-specific chromosome 19 miRNA cluster (C19MC) is essential in primate reproduction, development, and differentiation. miR-524-5p, a C19MC member, is highly homologous to the reprogramming miR-520d-5p; we also reported that miR-524-5p was expressed in iPSCs but not mesenchymal stem cells (MSCs). This study aimed to elucidate possible contributions of miR-524-5p to the reprogramming process.

**Methods:**

A miR-524-5p precursor was introduced into human fibroblast HFF-1 in the presence of OSKM, and the relative number of embryonic stem cell (ESC)-like colonies that stained positively with alkaline phosphatase (AP) and Nanog were quantified to determine reprogramming efficiency. A miR-524-5p mimic was transfected to MSCs to investigate the effects of miR-524-5p on TP53INP1, ZEB2, and SMAD4 expression by real-time polymerase chain reaction (PCR) and Western blot. Direct gene targeting was confirmed by luciferase activity. A phylogenetic tree of TP53INP1 was constructed by the Clustal method. Contribution of miR-524-5p to cell proliferation and apoptosis was examined by cell counts, BrdU, MTT, and cell death assays, and pluripotency gene expression by real-time PCR.

**Results:**

Co-expressing the miR-524 precursor with OSKM resulted in a two-fold significant increase in the number of AP- and Nanog-positive ESC-like colonies, indicating a role for miR-524-5p in reprogramming. The putative target, TP53INP1, showed an inverse expression relationship with miR-524-5p; direct TP53INP1 targeting was confirmed in luciferase assays. miR-524-5p-induced TP53INP1 downregulation enhanced cell proliferation, suppressed apoptosis, and upregulated the expression of pluripotency genes, all of which are critical early events of the reprogramming process. Interestingly, the TP53INP1 gene may have co-evolved late with the primate-specific miR-524-5p. miR-524-5p also promoted mesenchymal-to-epithelial transition (MET), a required initial event of reprogramming, by directly targeting the epithelial-to-mesenchymal transition (EMT)-related genes, ZEB2 and SMAD4.

**Conclusions:**

Via targeting TP53INP1, ZEB2, and SMAD4, miR-524-5p contributes to the early stage of inducing pluripotency by promoting cell proliferation, inhibiting apoptosis, upregulating expression of pluripotency genes, and enhancing MET. Other C19MC miRNAs may have similar reprogramming functions.

**Electronic supplementary material:**

The online version of this article (doi:10.1186/s13287-017-0666-3) contains supplementary material, which is available to authorized users.

## Background

Differentiated somatic cells can be reprogrammed into a pluripotent state by forced expression of a defined set of transcription factors, typically Oct4, Sox2, Klf4, and c-Myc (OSKM), to become induced pluripotent stem cells (iPSCs) [1]. The reprogramming process is thought to involve three phases (initiation, maturation, and stabilization), each of which is driven by a cascade of expression changes in specific sets of genes to give rise to fully or partially reprogrammed cells [2, 3]. Some important features of the early stage of reprogramming include increased proliferation, inhibition of apoptosis, acquisition of epithelial characteristics, and upregulation or activation of pluripotency-related genes [3]. Due to low reprogramming efficiencies, elucidating the molecular events that regulate each step of the reprogramming process has been challenging [4].

MicroRNAs (miRNAs), the negative regulators of gene expression, are often physically clustered on the human genome to permit co-regulation [5]. A number of chromosomally clustered miRNAs has been found to be specifically upregulated in pluripotent stem cells compared to mature differentiated cell types [5–7]. These include the miR-302/367, miR-17/92, and miR-200 clusters, and the chromosome 19 miRNA cluster (C19MC). The miR-302/367 cluster miRNAs are able to generate iPSCs [8, 9]. Alternatively, when co-expressed with the OSKM factors, members of the miR-302/367, miR-17/92, and miR-200 clusters are able to enhance reprogramming efficiency [10–12]. Some of these pluripotency-associated miRNAs have been shown to modulate somatic cell reprogramming at the early stages by inhibiting target genes in the cell cycle, mesenchymal-to-epithelial transition (MET), and the apoptosis pathways [5]. miRNA-302/367 promotes proliferation and accelerates G1 to S transition of the cell cycle by the targeting the Rb family and CDK1NA [13], whereas the miR-17/92 cluster enhances reprogramming efficiency by downregulating PTEN, a renowned tumor suppressor [11]. Moreover, both miR-302/367 and miR-200 clusters increase the kinetics of MET during reprogramming through blocking the epithelial-to-mesenchymal transition (EMT)-related genes TGFβR2 and ZEB1/ZEB2 [12, 14]. Despite a recent report on the involvement of miR-520d-5p (a member of the C19MC) in reprogramming [15], it is unclear if other C19MC miRNAs also contribute to inducing and regulating pluripotency.

C19MC, one of the largest miRNA gene clusters in the human genome, contains 46 highly homologous miRNA genes within a ~ 100-kb genomic region [16]. Importantly, C19MC is primate-specific and is predicted to play critical roles in primate reproduction, development, and differentiation [17], as reflected in restrictive expression of C19MC in only reproductive tissues and in pluripotent embryonic stem cells (ESCs) [7, 17, 18]. Our previous study has reported *en-bloc *C19MC activation in pluripotent stem cells but expression of only selective C19MC miRNAs in multipotent mesenchymal stem cells (MSCs) and a unipotent cell line [7]. Selective C19MC miRNA activation in cancer cells was also reported [19, 20] which showed similar expression patterns as in MSCs [7]. Bioinformatics analysis has further predicted that C19MC miRNAs may play a role in maintaining stem cell self-renewal and pluripotency by regulating the G1 to S transition phase of the cell cycle and the apoptosis pathway [7].

We report here that miR-524-5p, a C19MC member, was able to enhance OSKM-driven somatic reprogramming probably by promoting MET via targeting the EMT-associated genes ZEB2 and SMAD4. TP53INP1 was also identified as a miR-524-5p target that mediated enhanced cell proliferation and suppressed apoptosis, both of which are relevant to the early stage of reprogramming, establishing the contribution of a C19MC miRNA as an enhancer for cellular reprogramming.

## Methods

### Cell culture and maintenance of iPSCs

A human MSC line, WJ0706, derived from umbilical cord Wharton’s Jelly was obtained from and characterized at Cytopeutics Sdn. Bhd, Selangor, Malaysia (http://www.cytopeutics.com) according to standard procedures and with ethical clearance and subject consent [21]. Human adipose-derived MSCs, designated ASC-Inv and ASC Lonza, were purchased from Invitrogen (Carlsbad, CA, USA) and Lonza (Lonza, Verviers, Belgium), respectively. WJ0706, ASC-Inv, and ASC Lonza were maintained in Dulbecco’s modified Eagle’s medium (DMEM) nutrient mixture F-12 (DMEM/F12; Gibco, USA) with 10% fetal bovine serum (FBS; Gibco) at 37 °C and 5% CO_2_ in a humidified incubator. Human colon cancer HCT-15 and placental cell line HS799.PI were purchased from ATCC (Manassas, VA, USA), and the mouse embryonic fibroblast (MEF) cell line was purchased from EMD Millipore (Temecula, CA, USA). HCT-15, HS799.PI, and MEF were maintained in DMEM containing 10% FBS with 1% glutamax (Gibco). The foreskin fibroblast cell line, HFF-1 (ATCC), was maintained in DMEM containing 15% FBS with 1% glutamax (Gibco). 293FT cells (Invitrogen) were maintained in DMEM supplemented with 10% FBS, 0.1 mM MEM nonessential amino acids (NEAA), 6 mM l-glutamine, 1 mM MEM sodium pyruvate, and 500 μg/ml geneticin. ASC-IPSC and MH#1 were iPSC cell lines established from ASC-Inv and ASC Lonza, respectively, in our laboratory (S. Sugii, unpublished data). iPSC cells were maintained in DMEM/F12 containing 20% knockout serum replacement (KOSR; Invitrogen), 1% glutamax, 1× NEAA, 0.1% β-mercaptoenthanol, and 10 ng/ml basic fibroblast growth factor (bFGF; ReproCELL, Tokyo, Japan). The breast cancer cell line MCF-7 was kindly provided by Professor Y.M. Lim (Cancer Research Center, Universiti Tunku Abdul Rahman) and was maintained in RPMI medium (Gibco) with 10% FBS.

### Reprogramming of HFF-1 to iPSCs

The lentiviral vectors expressing Oct4, Sox2, KLF4, and c-MYC (Addgene, #20726, #20724, #20725, and #20723, respectively) and the inducible vector FUW-m2rtTA (Addgene, #20342) were prepared as previously described [22]. The HFF-1 cells were seeded in six-well plates and transduced with individual lentiviruses carrying human m2rtTA, OCT4, SOX2, KLF4, or C-MYC, with or without the miR-524 precursor (System Biosciences, Mountain View, CA, USA). On day 5 after transduction, 25,000 cells/well of a 12-well plate were transferred in triplicate wells onto MEF feeder cells. On the next day, the culture medium was switched from HFF-1 growth medium to human iPSC growth medium containing 2 μg/ml doxycycline (Dox). Typically, human ESC-like colonies started to appear around day 7. The ESC-like colonies were stained with alkaline phosphatase (AP) live stain (Life Technologies, Eugene, OR, USA) on day 14, and the Nanog live stain SmartFlare™ (EMD Millipore, Temecula, CA, USA) on day 18. Images of colonies with ESC-like morphology that were also staining positively with AP were obtained on day 14 using a fluorescence microscope (Carl Zeiss, USA); images of Nanog-positive colonies were similarly obtained on day 18. In comparing the AP and Nanog images, only the ESC-like and AP and Nanog double-positive colonies were counted. Reprogramming efficiency was calculated as the fraction of the number of ESC-like/AP^+^Nanog^+^ colonies formed over the total number of input cells in triplicate wells and in three independent experiments.

### miRNA and mRNA expression analysis

Total RNA was isolated from the cell lines by using the MiRNeasy Mini Kit (Qiagen, Valencia, CA, USA) according to the manufacturer’s instructions. For miR-524-5p quantification using the TaqMan microRNA assay (Applied Biosystems, Foster City, CA, USA), first-strand cDNA synthesis was carried out using a TaqMan MicroRNA Reverse Transcription kit (Applied Biosystems) and miR-524-5p-specific primer (Cat. no. 4427975, Applied Biosystems) according to the manufacturer’s protocol. Real-time polymerase chain reaction (PCR) was carried out with TaqMan Universal PCR Master Mix (Applied Biosystems) and TaqMan MicroRNA Assay Mix on a thermal cycler (Rotor-Gene Q, Qiagen). For gene expression at the mRNA level, first-strand cDNA was generated using the SuperScript III (Invitrogen) according to the manufacturer's instructions. The SYBR Select Master Mix kit (Applied Biosystems) was used for real-time PCR quantification, and the real-time reaction was carried out in a Rotor-Gene Q. RNU6B and GAPDH were used as the normalization controls for miRNA and mRNA assays, respectively. The primers for mRNA quantification are listed in Additional file 1: Table S1. The expression of miRNAs and mRNAs was calculated based on the ΔΔC_T_ method. All experiments were preformed in triplicate, and three or more independent experiments were performed to obtain the results presented.

### Determination of absolute copy number of mature miRNAs

The copy number of miR-524-5p was determined as previously described [7]. In brief, a synthetic mature miR-524-5p oligonucleotide (Integrated DNA Technologies IDT, Coralville, IA, USA) was serially diluted 10-fold to final concentrations between 200 nM and 0.02 pM. The serially diluted synthetic miRNA aliquots were reverse-transcribed and subjected to real-time PCR analysis concurrently with the sample RNAs. Serially diluted standard-curve samples were included on each plate of the miRNA TaqMan assays to convert the cycle threshold (C_t_) values of each sample into the corresponding number of miRNA copies in each cell, assuming that each cell contained 15 pg total RNA, as previously described [23]. C_t_ values ≥ 35 indicated expression levels too low for accurate analysis, and were considered as an undetectable expression level. The cut-off threshold of miRNA expression was, therefore, standardized at C_t_ < 35.

### Transient transfection

MirVana miR-524-5p mimic (Cat. no. 4464066) and negative control (NC; Cat. no. 4464058) were designed and synthesized by Ambion (Foster City, CA, USA), whereas the ON-TARGETplus SMARTpool siTP53INP1 (Cat. no. L-016159-00-0005, GE Dharmacon, CO, USA) containing a mixture of four SMART selection-designed small interfering RNAs (siRNAs) targeting the human TP53INP1 gene was used. For transfection of the colorectal cancer cell line HCT-15, the cells were seeded onto six-well plates at a density of 2.5 × 10^5^ cells/well and were transiently transfected using Lipofectamine™ 2000 Reagent (Invitrogen) according to the manufacture’s protocol. For WJ0706 transfection, the cells were seeded at a density of 9.5 × 10^4^ cells/well in a six-well plate, and transfection was performed using Lipofectamine™ RNAiMAX Reagent (Invitrogen). The cells were harvested for analysis 48 h post-transfection.

### Protein extraction and Western blotting

Total protein lysates were prepared using the RIPA lysis buffer (Nacalai Tesque, Kyoto, Japan). Proteins at 50 μg per lane were separated by 8% sodium dodecyl sulfate-polyacrylamide gel electrophoresis and transferred to a nitrocellulose membrane (Amersham ECL, Freiburg, Germany). The membrane was then blotted with polyclonal antibodies against TP53INP1 (1:1000, ab9777, Abcam, Cambridge, UK), SMAD4 (1:500, ab137861, Abcam), β-actin (1:1000, ab8227, Abcam), or monoclonal antibody ZEB2 (1:500, sc-271984, Santa Cruz, CA, USA), followed by horseradish peroxidase-conjugated secondary antibodies. Visualization was achieved using chemiluminescence (GE Healthcare Life Sciences, Piscataway, NJ, USA).

### Phylogeny tree analysis

Phylogenetic tree alignment of the 3’-untranslated region (UTR) of the TP53INP1 transcript sequences in different species was generated by the Clustal method using the DNAstar software (Madison, WI, USA).

### Luciferase assays

PCR products of fragments covering the predicted miR-524-5p binding sites in the 3’-UTR of the TP53INP1, ZEB2, and SMAD4 transcripts were cloned at the 3’-end of firefly luciferase gene in the dual reporter vector pmirGLO (GenBank accession FJ376737; Promega, Madison, WI, USA) at the SacI and XbaI restriction sites. Mutations of the miRNA seed sequences were performed using the QuikChange® Lightning Site-Directed Mutagenesis kit (Agilent Technologies, Santa Clara, CA, USA) as recommended by the supplier. The mutations were confirmed by sequencing. Sequences of the PCR primers used are shown in Additional file 2: Table S2. Co-transfection into HCT-15 cells was performed by using Lipofectamine™ 2000 (Invitrogen) according to the manufacture’s protocol. A validated miR-524-5p mimic, or mimic negative control (NC; Ambion), was used in co-transfection with the luciferase wild-type or mutant constructs. Luciferase assays were performed 48 h post-transfection using the Dual-Luciferase Reporter 1000 Assay kit (Promega). Transfection experiments were performed in two or more independent experiments with quadruple transfections each.

### Cell proliferation assays

WJ0706 cells were transfected with miRNA mimic miR-524-5p, miRNA NC, or TP53INP1 siRNA (siTP53INP1). After 48 h incubation, cells were seeded in six-well plates at a density of 1 × 10^4^ cells/well. After 2, 4, and 6 days post-transfection, the cells were trypsinized and stained with trypan blue (Gibco Invitrogen). The number of viable and dead cells was counted using a Neubauer counting chamber. For 5-bromo-2’-deoxyuridine (BrdU) measurements, 48 h post-transfection, cells were seeded in 96-well plates at a density of 5000 cells/well for 24 h. Cell proliferation was measured using the BrdU cell proliferation assay kit (Cell Signaling Technology, Denver, MA, USA) according to the manufacturer’s instructions. BrdU incorporation was monitored at 450 nm. Data presented are from three independent experiments, and the results of the treated cells were normalized with the untreated control cells.

### MTT assays for cell viability

MTT (3-(4,5-dimethylthiazol-2-yl)-2,5-diphenyltetrazolium bromide; Sigma Chemical Co., St. Louis, MO, USA) was used to quantify cell survival from H_2_O_2_-induced oxidative stress. Briefly, after 48 h hours post-transfection with miRNA or NC mimic or siTP53INP1, the transfected WJ-MSC cells were treated with 200 μM H_2_O_2_ for 2 h. Subsequently, the cells were trypsinized and seeded in 96-well plates at a density of 5000 cells/well and cultured for 24 to 96 h, followed by the addition of 10 μl 5 mg/ml MTT to each well and incubation for 2.5 h. The reaction was stopped by adding 100 μl dimethyl sulfoxide. Absorbance at 570 nm was determined using a plate reader.

### Histone/DNA ELISA for detection of apoptosis

The Cell Death Detection enzyme-linked immunosorbent assay (ELISA) plus kit (Roche Diagnostics, Penzberg, Germany) was employed to quantitatively detect histone-associated DNA fragments in mono- and oligonucleosomes according to the manufacturer’s protocol. Briefly, after 48 h post-transfection, cells were trypsinized and seeded in 96-well plates at a density of 5000 cells/well and cultured for 24 h. The cells were treated with 200 μM H_2_O_2_ for 6 h. After treatment, the cytoplasmic histone/DNA fragments from the cells were extracted and incubated in microtiter plate modules coated with an antihistone antibody. Subsequently, peroxidase-conjugated antiDNA antibody was used for the detection of immobilized histone/DNA fragments followed by color development with ABTS substrate for peroxidase. The spectrophotometric absorbance of the samples was determined at 405 nm.

### Prediction of miRNA target genes

The 3’-UTR sequences of putative target genes were retrieved from the UCSC genome browser (http://genome.ucsc.edu). miRNA:mRNA interactions were predicted using TargetScan, miRanda, and DIANA-microT. Functions of the target genes were elucidated through the Kyoto Encyclopedia of Genes and Genomes (KEGG) pathway.

### Statistical analysis

Data were analyzed by paired Student’s *t* test (two-tailed distribution) comparing the differences of expression levels between treatment and nontreatment cells. Statistical significance was accepted at *p* < 0.05.

## Results

### miRNA-524-5p enhances reprogramming

**Fig. 1:**
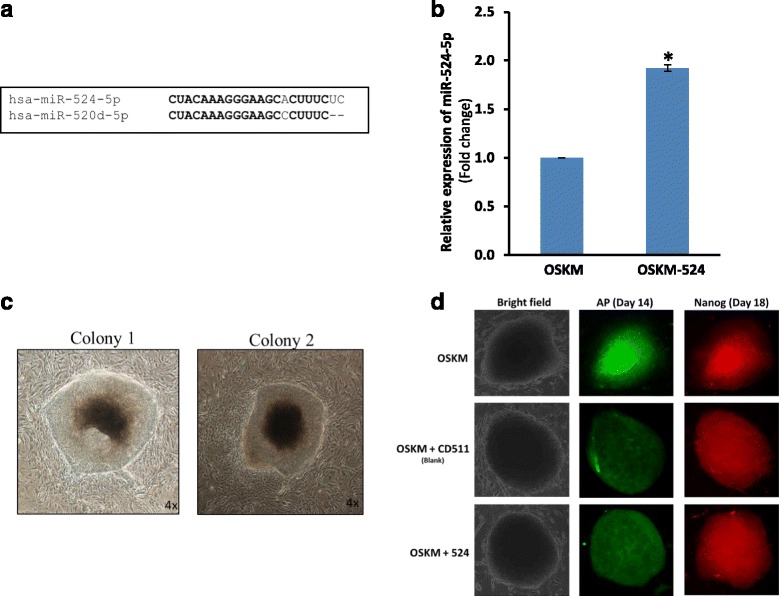
Overexpression of mir-524 precursor promotes OKSM-driven iPSC generation at the early stage of induction. **a** High degree of sequence homology (*bold letters*) between miR-524-5p and miR-520d-5p. **b** Upregulated miR-524-5p levels in OSKM/mir-524 co-transduced relative to OSKM-transduced HFF-1 cells. *p < 0.05. **c** ESC-like morphology of two representative colonies formed at passage 1. **d** ESC-like alkaline phosphatase (AP)-positive colonies on day 14 that co-stained with Nanog on day 18 post-transduction with OSKM only, or OSKM in combination with either the blank vector CD511 or with a mir-524 precursor construct

We previously showed in miRNA microarray and miRNA copy number analyses that miR-524-5p was expressed abundantly in pluripotent stem cells, including ESCs and iPSCs, whereas miR-524-5p expression was undetected or detected at low levels in the MSC cell lines from which the iPSCs were derived [7]. Furthermore, miR-524-5p shares 19/20 nucleotides with miR-520d-5p (Fig. 1a), suggesting identical biological functions for both miRNAs. Recent studies have indicated the ability of miR-520d-5p for converting cancer cells into iPSC-like cells [15, 24]. Hence, we hypothesized that miR-524-5p may also play an important role in reprogramming in iPSCs.

To test if miR-524-5p also enhances reprogramming in the presence of other known reprogramming factors, the lentiviral vector-based pCDH-mir-524 construct encoding the mir-524 precursor was used to determine the effect of miR-524-5p on iPSC induction in human fibroblast HFF-1 cells (Fig. 1b–d). HFF-1 cells were transduced with pCDH-mir-524 together with the Dox-inducible lentiviral vectors each carrying the human cDNAs encoding one of the four transcription factors OCT4, SOX2, c-MYC, and KLF4 (OSKM), and a constitutively active lentivirus transducing the reverse tetracycline transactivator FUW-m2rtTA (see Methods). The blank vector pCDH-CD511 was also included as a transduction control. In the OSKM/mir-524 co-transduction, and in the ESC-like colonies formed (see below), the miR-524-5p level was determined to be double that of the endogenous miR-524-5p levels of ~ 11.9 × 10^4^ copies/cell (data not shown) found in colonies of the OSKM-transduced cells (Fig. 1b). After transduction, the cells were cultured under standard conditions for human iPSC induction but in the presence of Dox, and the transduced cells were monitored daily for morphological changes. ESC-like colonies began to form on day 7 which, when passaged on day 30 and cultured under Dox-free conditions, showed clear and round borders (Fig. 1c). Furthermore, these colonies showed AP staining on day 14 and Nanog at day 18 culturing under Dox-dependent medium (Fig. 1d). Colonies that displayed ESC-like morphology and that were both AP- and Nanog-positive were considered *bona fide* iPSC colonies (Fig. 1c and d). In each of the three independent experiments, the total number of ESC-like and AP^+^Nanog^+^ colonies observed varied between three to six in the triplicate wells of the 12-well plate transduced with OSKM alone, or OSKM with the blank vector CD511, and from seven to twelve colonies on OSKM/mir-524 transduction (Table 1). Taken together, OSKM/mir–524 co-transduction generated a total of 27 ESC-like/AP^+^Nanog^+^ colonies in the three independent transduction experiments, with a calculated reprogramming efficiency of 0.012%, and was 2.25-fold that of OSKM or OSKM/CD511 transduction, which was within the range of reprogramming efficiencies reported by others [25, 26]. The data thus support the notion that miR-524 enhanced OKSM-induced reprogramming of HFF-1 fibroblast cells.

**Table 1:** Number of ESC-like and AP^+^Nanog^+^ colonies obtained on OSKM/mir-524 co-transduction of HFF-1 cells

Transduction	Colonies in experiment (*n*)			Total colonies (*n*)	SD	Reprogramming efficiency (%)
	1	2	3			
OSKM only	4	3	6	12	1.53	0.005
OSKM + CD511	6	4	3	12	1.53	0.005
OSKM + mir-524	8	7	12	27*	2.65	0.012*

ESC-like and AP^+^Nanog^+^ colonies in triplicate wells in a 12-well plate were counted under a microscope as described in Methods. Reprogramming efficiency was calculated as the total number of AP^+^Nanog^+^ colonies generated from the total number of transduced HFF-1 cells in three independent experiments. *p < 0.05 compared with OSKM only. *AP* alkaline phosphatase, *ESC* embryonic stem cell, *SD* standard deviation

### Bioinformatics analysis of miRNA-524-5p and predicted target mRNA interactions

**Fig. 2:**
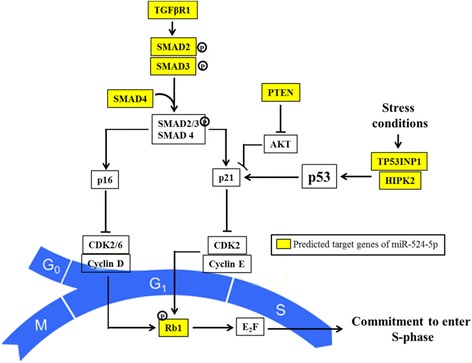
Predicted miR-524-5p-targeted genes regulate the G1 to S transition phase of the cell cycle. The predicted target genes were derived by interrogation of a variety of miRNA target prediction algorithms including the TargetScan, miRanda, and DIANA-microT. Putative miR-524-5p target genes are shown in *yellow boxes*

In our previous work, we have described the bioinformatics analysis of C19MC miRNAs, including the most significantly enriched gene ontology terms associated with biological process and molecular functions and the KEGG pathways [7]. In the same study, our data showed that C19MC could play an important role in regulating stemness. Since cell cycle, more critically the G1-to-S transition phase, is an important feature of the regulation of stem cell self-renewal [13, 27] we focused in this work on determining possible functions of miR-524-5p in relation to the G1-S phase of the cell cycle. Based on the earlier bioinformatics analysis [7], eight predicted G1-to-S transition-related genes, namely TGFβR1, Smad2/3/4, Rb1, PTEN, HIPK2, and TP53INP1, were identified to be targeted by miR-524-5p (Fig. 2).

Repression of the PI3K/PKB/Akt/mTOR and TGFβ pathways, such as PTEN, p21/CDK1NA, TGFβR1, and SMAD2/3/4, has been reported to promote self-renewal and proliferation by blocking the G1 to S transition boundary of the cell cycle [13, 28–30]. Furthermore, p53, a pro-apoptotic, antiproliferative, and antioxidant regulator, is indirectly regulated by miR-524-5p through targeting TP53INP1 and HIPK2 (Fig. 2). Suppression of HIPK2 could inhibit p53 expression [31] whereas TP53INP1 is a major mediator of p53-driven responses to oxidative stress [32]. Besides p53, miR-524-5p was also predicted to target a member of the Rb family, RB1, an event that regulates the cell cycle by enhancing G1 to S transition and proliferation. TP53INP1 plays important roles not only in reprogramming by regulating p53 [33, 34] but also in cancer stem cells in which TP53INP1 deficiency results in increased self-renewal and acquisition of cancer stem cell phenotype [35, 36]. Thus, the possible correlation between miR-524-5p and TP53INP1 was further investigated.

### TP53INP1 is a direct target of miR-524-5p

**Fig. 3:**
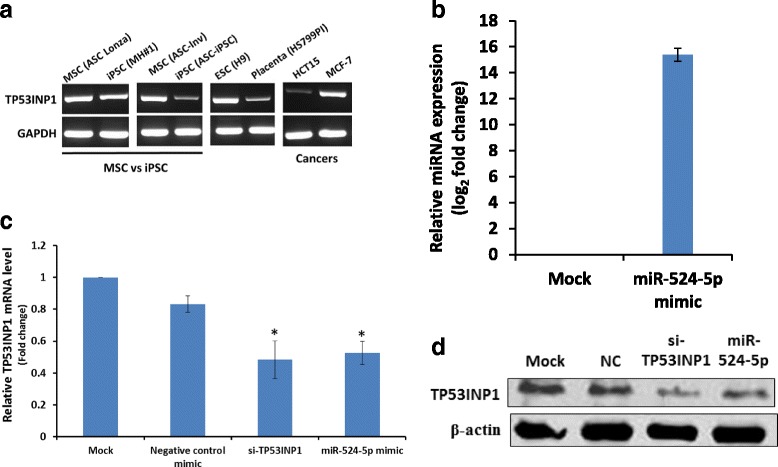
Inverse relationship between expression of miR-524-5p and TP53INP1. **a** Expression of TP53INP1 in different cell lines determined by 25 cycles of RT-PCR. iPSC (MH#1) and iPSC (ASC-iPSC) were derived from the adipose-MSC cell lines, ASC Lonza and ASC-Inv, respectively. ESC (H9), placenta (HS799.PI), colon cancer (HCT-15), and breast cancer (MCF-7) cell lines were included for comparison and as controls. **b** Efficient transfection and overexpression of the miR-524-5p mimic in HCT-15 cells. **c**,**d** Effects of miR-524-5p overexpression on TP53INP1 expression. A miR-524-5p mimic was transfected into HCT-15 cells for 48 h before the cells were harvested for 40 cycles of real-time RT-PCR (**c**) or Western blot analysis (**d**) to determine TP53INP1 expression. As a control, a TP53INP1 siRNA (siTP53INP1) was also used in the transfection. *p < 0.05, relative to mock transfection. *ASC* adipose-derived stem cell, *ESC* embryonic stem cell, *iPSC* induced pluripotent stem cell, *MSC* mesenchymal stem cell, *NC* negative control

To investigate the relationship between miR-524-5p and TP53INP1, endogenous TP53INP1 expression in MSC and iPSC lines was first established by RT-PCR (Fig. 3a). TP53INP1 was found to be expressed in both MSCs and iPSCs, albeit at higher levels in MSCs than in the derived iPSCs. Interestingly, our previous study has shown that miR-524-5p was abundantly expressed in iPSCs whereas miR-524-5p expression was undetected or detected at very low levels in MSC cell lines [7], suggesting an inverse correlation between the expression of miR-524-5p and TP53INP1 and negative regulation of TP53INP1 by miR-524-5p. ESCs and placenta, which comprehensively express all the C19MC miRNAs and, therefore, miR-524-5p [7], also expressed TP53INP1, and in higher levels in ESCs. Surprisingly, the two cancer cell line controls, the colorectal HCT-15 and the breast cancer MCF-7, also expressed TP53INP1 to different levels (Fig. 3a). Notably, all these cell lines, except for the placenta HS799.PI cells, also expressed various levels of miR-524-5p [7], but correlation could not be made between the TP53INP1 and miR-524-5p expression levels. The observation of the lack of inverse correlation between miR-524-5p and TP53INP1 in some cell lines suggests that, besides regulation by miR-524-5p, other factors are involved in regulating TP53INP1 expression, particularly in cancer cells (see Discussion).

When HCT-15 cells were transfected with a miR-524-5p mimic to achieve a ~ 16-fold upregulation of the miR-524-5p level 48 h post-transfection (Fig. 3b), the TP53INP1 mRNA and protein levels were assayed in qRT-PCR and Western blots (Fig. 3c and d). As controls, a miRNA negative transfection control (NC) and a siRNA to knockdown TP53INP1 expression were also included. Similar to siRNA-mediated TP53INP1 suppression, forced overexpression of miR-524-5p significantly downregulated TP53INP1 at both the mRNA and protein levels compared with the mock control (Fig. 3c and d), further confirming a reverse relationship between miR-524-5p and TP53INP1 expression. The data further suggested that miR-524-5p regulated TP53INP1 expression probably via downregulation of the TP53INP1 transcript.

**Fig. 4:**
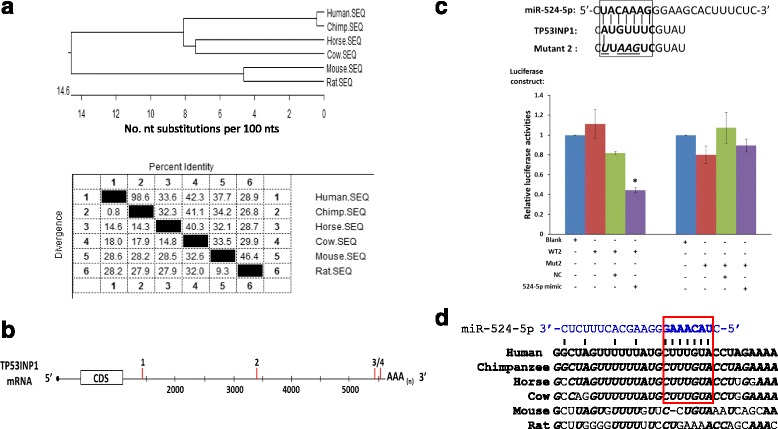
Direct TP53INP1 targeting by and possible co-evolution with miR-524-5p. **a** Phylogenetic tree alignment (*top panel*) and sequence comparison (*bottom panel*) of the 3’-UTR of TP53INP1 ortholog transcript sequences in different species. **b** Identification of four putative miR-534-5p target sites (*red vertical bars*) in the 3’-UTR of the human TP53INP1 transcript. **c** Experimental validation of miR-524-5p targeting TP53INP1 in luciferase assays. In the *top panel*, alignment of miR-524-5p with the putative target site 2 (boxed and in bold letters) in the 3’-UTR of the TP53INP1 transcript (see text) is shown, so are the mutations (in italics and underlined) in the luciferase construct Mut2. HCT-15 transfected with the wild-type (*WT2*) or the mutant (*Mut2*) luciferase constructs alone, or in the presence of the miR-524-5p mimic or a validated negative control (*NC*), was performed before luciferase assays. The data shown were derived from two independent experiments in triplicate. *p < 0.05. **d** Alignment of sequences around the active target site 2 of TP53INP1 in different species. The miR-524-5p miRNA sequence is shown in *blue* above the TP53IPN1 sequences. The targeted core sequences are underlined and shown in bold. Similar nucleotides relative to the human gene are shown in italics and in bold letters. *CDS* coding sequence.

Interestingly, when the TP53INP1 transcript sequences of various species were compared, TP53INP1 sequences of the primate (human and chimpanzee) showed a tight clustering with a high sequence homology of 98.6% and late evolutionary emergence in comparison with other mammalian orthologs (Fig. 4a). Keeping in mind that miR-524-5p belongs to the primate-specific C19MC cluster, the observation may hint at primate-specific co-evolution of the miR-524-5p and TP53INP1 gene sequences. The long 4521-bp 3’-UTR of the TP53INP1 transcript (NM_033285) encompasses four putative miR-524-5p-targeted sites, each with a six- to seven-nucleotide seed sequence alignment with the TP53INP1 sequence at nucleotide (nt) positions 1461–1466, nt 3397–3403, nt 5450–5455, and nt 5530–5536 of the transcript (Fig. 4b). Target sites 1, 3, and 4 did not show appreciable downregulation of luciferase activities in luciferase assays using the pmirGLO vector (data not shown). On the other hand, luciferase construct of target site 2, designated as WT2, when co-transfected with the miR-524-5p mimic resulted in a reduction to ~ 40% of luciferase activity relative to that in the control cells transfected with the blank vector (Fig. 4c). Specific targeting was confirmed with transfection of the mutated target site 2 in the Mut2 construct, which did not show appreciable effects on luciferase activities (Fig. 4c). Echoing possible co-evolution of miR-524-5p and TP53INP1 suggested above, the active target site 2 also showed identical sequences between the two primate genes, but not with other mammalian orthologs (Fig. 4d). Taken together, luciferase assays confirmed that miR-524-5p directly targets TP53INP1 to downregulate TP53INP1 expression, and that the miRNA and target gene may have co-evolved late in the evolution of the primate.

### miR-524-5p regulates processes relevant to reprogramming

**Fig. 5:**
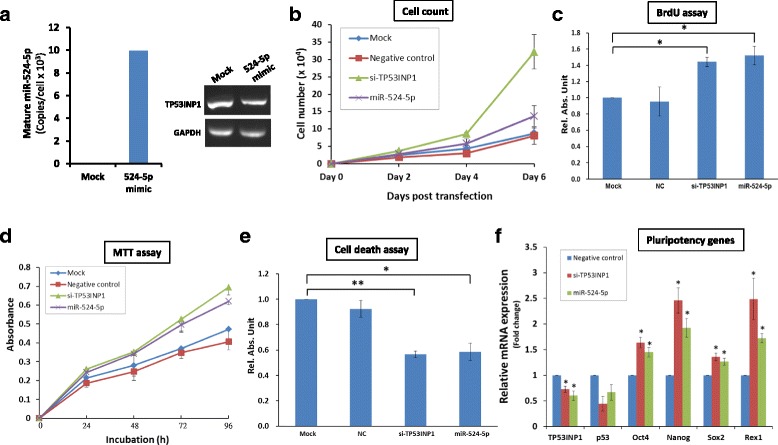
miR-524-5p regulates critical features of cellular reprogramming via targeting TP53INP1. **a** Endogenous transcript levels of miR-524-5p and TP53INP1 in WJ0706 cells. Before (mock) or after transfection of a miR-524-5p mimic, WJ0706 cells were harvested 48 h post-transfection for analysis; miR-524-5p copy number was determined by Taqman qRT-PCR (*left panel*); TP53INP1 transcript level was determined by direct RT-PCR (*right panel*). **b**–**f** The miR-524-5p mimic-transfected WJ0706 cells were subjected to assays to determine cell proliferation (**b**,**c**), cell viability (**d**), apoptosis after oxidative stress induced with 200 μM H_2_O_2_ (**e**), and expression of selected pluripotency genes (**f**). All the experiments also included mock transfection with a negative control (*NC*) miRNA mimic, or a TP53INP1 siRNA (siTP53INP1) included as controls. For effects on cell proliferation, cell counts at different days post-transfection (**b**), or by BrdU ELISA measurements (**c**) were performed. For apoptosis assay (**d**,**e**), cell viability 2 h after incubation with H_2_O_2_ was determined by the MTT assay (**d**), or the histone-associated DNA fragments of apoptotic cells were quantified by ELISA assay 6 h after H_2_O_2_ treatment (**e**). **f** Expression of pluripotency genes in the transfected cells was analyzed by real-time RT-PCR 48 h post-transfection. Relative absorbance unit and mRNA level were determined as the relative absorbance units and expression, respectively to the value of the mock and negative control experiments, respectively, which was set as 1.0. *p < 0.05, **p < 0.01

**Fig. 6:**
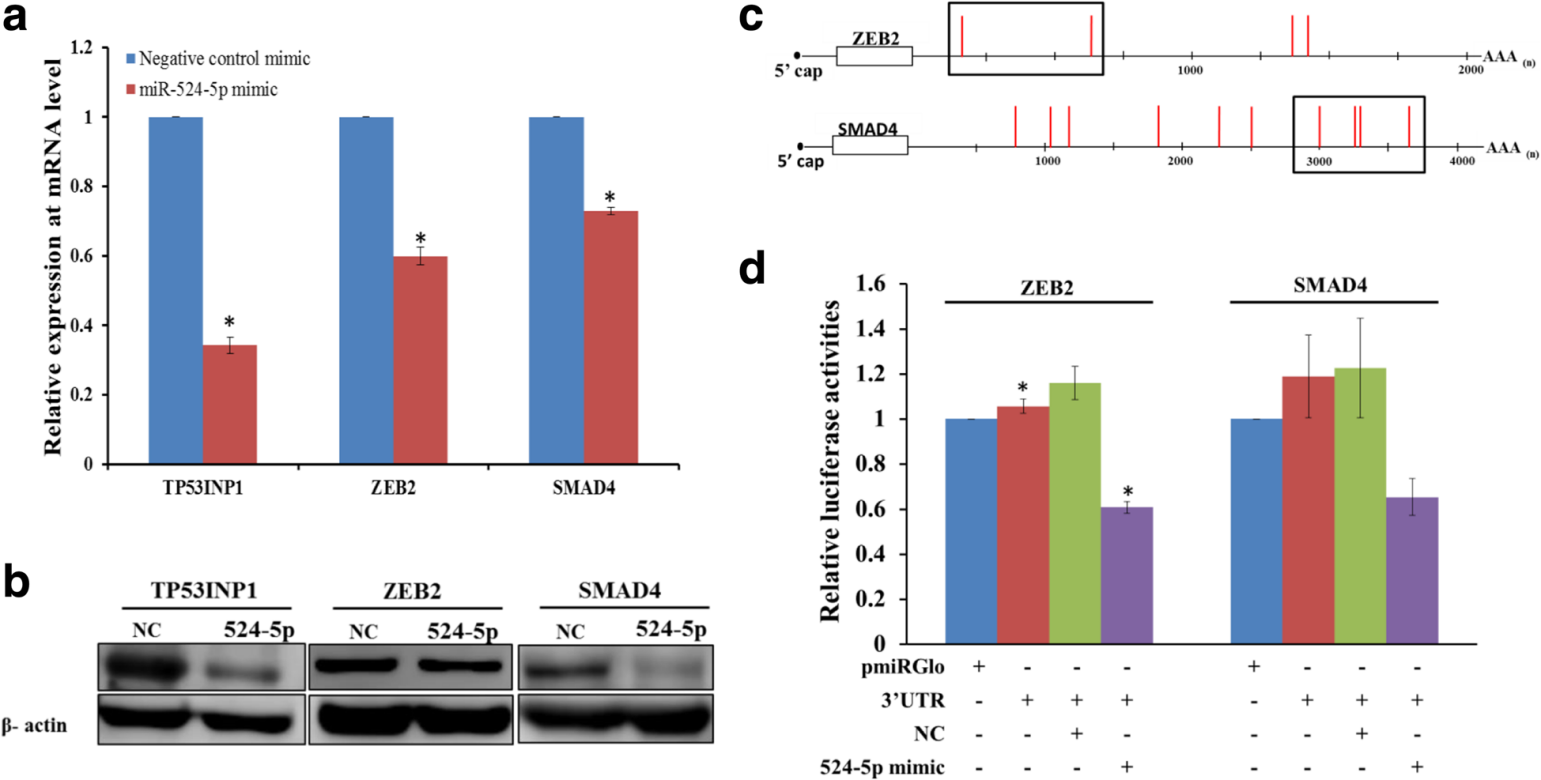
EMT-related genes ZEB2 and SMAD4 are direct targets of miR-524-5p. **a**,**b** Effects of miR-524-5p overexpression on the expression of the EMT-related genes. A miR-524-5p mimic was transfected to WJ0706 cells for 48 h before the cells were harvested for analysis. The analysis of the expression of ZEB2 and SMAD4 by real-time RT-PCR (**a**) and Western blot analysis (**b**). In both experiments, TP53INP1 was included as a control. **c**,**d** Experimental validation of miR-524-5p targeting of ZEB2 and SMAD4 in luciferase assays. Based on the predicted miR-524-5p binding sites (*red vertical lines*) in the 3’-UTRs of the ZEB2 and SMAD4 transcripts, a 3’-UTR luciferase construct of each gene was generated (*boxed*) (**c**). The blank pmiRGlo and 3’-UTR luciferase constructs were transfected alone, or in the presence of the miR-524-5p mimic, or a validated negative control (*NC*) in colon cancer cell line HCT-15 prior to luciferase assays (**d**). *p < 0.05, relative to the pmiRGlo-only control

The cellular reprogramming process is a dynamic and tightly controlled process driven by cascades of cellular and molecular events. Two such events that occur at the early stages of reprogramming are accelerated proliferation rates and suppression of apoptosis [3]. Since a Wharton’s Jelly MSC 0706 (WJ0706) cell line was used in subsequent experiments, the endogenous expression levels of both miR-524-5p and TP53INP1 in WJ0706 cells were first determined based on transcript copy number and RT-PCR, respectively (Fig. 5a). The results indicated that miR-524-5p was undetectable in WJ0706 cells (Fig. 5a, left panel, the mock control), whereas the expression level was upregulated to about 10,000 copies/cell 48 h post-transfection of the miR-524-5p mimic (Fig. 5a, left panel, 524-5p mimic). On the other hand, the endogenous TP53INP1 transcript level was high in WJ0706 (Fig. 5a, right panel, mock control); however, the TP53INP1 transcript level was reduced 48 h post-transfection of the miR-524-5p mimic (Fig. 5a, right panel), which also echoed the miR-524-5p-suppressed TP53INP1 transcript and protein levels in HCT-15 cells (Fig. 3c and d; see also Fig. 6b, left most panel below). Next, to examine whether miR-524-5p expression influences the cell proliferation rate, WJ0706 cells were transfected with the miR-524-5p mimic and in vitro cell proliferation assays were performed. The data showed enhanced cell proliferation rates 3 to 4 days post-transfection, correlating with results of the TP53INP1 knockdown (Fig. 5b). The miR-524-5p-transfected cells also showed ~ 50% significantly higher BrdU incorporation compared to the mock control as in the experiment with TP53INP1 knockdown by siRNA transfection, which also significantly increased BrdU incorporation by 40% (Fig. 5c). Hence, miR-524-5p enhances cell proliferation which may partially be due to TP53INP1 downregulation.

Reprogramming is a stressful process that increases cellular levels of reactive oxygen species (ROS), resulting in the activation of apoptosis [37]. To analyze the effects of miR-524-5p on cell viability in response to oxidative stress, miR-524-5p-transfected cells were exposed to 200 μM H_2_O_2_ for 2 h and the metabolic activities in the treated cells were measured by MTT assay. The results indicated that transfection of the miR-524-5p mimic suppressed the damaging effects of H_2_O_2_-induced oxidative stress on cell viability, and the protection was likely via TP53INP1 since TP53INP1 knockdown also resulted in similar protection effects (Fig. 5d). The effects of miR-524-5p on oxidative stress-induced apoptosis was further determined by ELISA quantification of the histone-associated DNA fragments in mono- and oligonucleosomes produced during nuclear DNA denaturation of apoptotic cells (Fig. 5e). The result showed ~ 40% significant reduction of nucleosome production in the miR-524-5p- or siTP53INP1-transfected cells compared to the mock control, further indicative of miR-524-5p suppression of apoptosis via TP53INP1.

The transition from the initiation to the maturation phase of the reprogramming process is also highlighted by upregulation of pluripotency-associated genes, including Oct4, Nanog, Sox2, and Rex1 [2, 3], which were assayed in miR-524-5p mimic- and siTP53INP1-transfected cells (Fig. 5f). Overexpression of miR-524-5p downregulated TP53INP1 mRNA levels as anticipated, as did the transfection of siTP53INP1. Furthermore, expression of the p53 gene, which acts downstream of TP53INP1 (Fig. 2), was concurrently downregulated, also as anticipated [32]. The data clearly showed that both overexpression of miR-524-5p and TP53INP1 knockdown upregulated the expression of all four pluripotency genes tested in the transfected cells (Fig. 5f). Hence, the results support that miR-524-5p negatively regulates TP53INP1, which in turn regulates p53, to upregulate expression of pluripotency genes. Taken together, our results demonstrated involvement of miR-524-5p in events relevant to reprogramming, namely promotion of cell proliferation, suppression of oxidant-induced apoptosis, and upregulation of pluripotency-associated genes via targeting and downregulation of the TP53INP1 transcript in the p53 signaling pathway.

### miR-524-5p promotes MET required for initiating reprogramming by downregulating EMT-related genes

On reprogramming, besides promotion of cell proliferation, suppression of oxidant-induced apoptosis, and upregulation of pluripotency-associated genes, MET is also an essential initiation step for progression towards pluripotency [3]. Our bioinformatics analysis revealed identical seed sequence and high degree of sequence homology between miR-524-5p and miR-520d-5p (Fig. 1a). Importantly, miR-520-5p has previously been reported to inhibit expression of the EMT-related gene TWIST1 [38]. Hence, we hypothesized that miR-524-5p may also enhance MET by targeting the EMT-associated genes that were predicted to be targeted by miR-524-5p. To test this hypothesis, a miR-524-5p mimic was first transiently transfected into WJ0706 cells, which do not express miR-524-5p, and the mimic transfection resulted in an increase of the miR-524-5p to ~ 10,000 copies/cell 48 h post-transfection (see above and Fig. 5a, left panel). It was also noted that the transfected miR-524-5p expression level in WJ0706 was lower than that in HCT-15 (Fig. 3b), suggesting different efficiencies in transient miRNA transfection of cancer and stem cells. Nevertheless, the upregulated miR-524-5p level in WJ0706 was sufficient to downregulate the expression of its target genes including TP53INP1, ZEB2, and SMAD4 (Fig. 6a).

The expression of the predicted EMT-related target genes, including TGFβR1, SMAD2, SMAD3, SMAD4, ZEB1, ZEB2, and TWIST1, was determined by RT-PCR, which showed that only ZEB2 and SMAD4 expression were downregulated 48 h post-transfection with the miR-524-5p mimic (data not shown). miR-524-5p downregulation of endogenous expression of ZEB2 and SMAD4 was confirmed in quantitative real-time RT-PCR and Western blot analysis (Fig. 6a and b). Compared to transfection with the negative control mimic, forced expression of miR-524-5p significantly inhibited endogenous mRNA expression of ZEB2 and SMAD4 by almost 40% and 30%, respectively (Fig. 6a). The protein levels of ZEB2 and SMAD4 were also diminished by miR-524-5p mimic (Fig. 6b). Thus, these data suggested negative regulation of expression of ZEB2 and SMAD4 by miR-524-5p, which was confirmed by demonstration of direct miRNA targeting in luciferase assays (Fig. 6c and d). The 3’-UTR construct of ZEB2 carried the two 5’ predicted target sites whereas the SMAD4 construct carried a cluster of four putative target sites (Fig. 6c). The ZEB2 or SMAD4 3’-UTR construct was individually transfected into HCT-15 cells alone, or in the presence of miR-524-5p mimic or a NC, which resulted in 40% and 35% reduction of luciferase activity, respectively, when co-transfected with the miR-524-5p mimic (Fig. 6d). Taken together, the results confirmed that miR-524-5p targeted and repressed the expression of the EMT-associated genes ZEB2 and SMAD4, which may have a direct bearing on the initial phase of establishing pluripotency.

## Discussion

Emerging evidence has indicated that miRNAs play some crucial roles in somatic cell reprogramming, self-renewal, and differentiation [5]. Overexpression of miR-520d-5p alone has been reported to successfully convert hepatoma cells into iPSC-like cells [15]. Since both C19MC members including miR-520d-5p and miR-524-5p are highly homologous in sequences and share the same seed sequence (Fig. 1a), the two miRNAs may share similar biological functions. It was first noted that miR-524-5p alone was unable to reprogram the normal fibroblast cells tested (data not shown). However, miR-524-5p was effective in enhancing the OSKM factor-driven reprogramming process. By targeting TP53INP1, miR-524-5p was shown to enhance proliferation and suppress apoptosis (Fig. 5a–d), both of which are early crucial events for reprogrammable cells to enter subsequent phases of activation or upregulation of pluripotency genes in the progression of the reprogramming process [2, 3]. Furthermore, miR-524-5p was shown to target and downregulate expression of the EMT-related genes ZEB2 and SMAD4 and, hence, promoting MET, which is also an essential initial event of reprogramming [2, 3]. Others have reported that introduction of multiple members of the miR-302/367 family was able to rapidly and efficiently reprogram fibroblasts into iPSCs with or without other reprogramming factors [9, 10]. The miR-302/367 cluster enhances reprogramming efficiency by increasing cell division rate [13] and suppressing apoptosis [39], as well as promoting epigenetic reactivation of pluripotency genes [40], as shown here for miR-524-5p. In addition, miR-138 suppresses expression of p53 and its downstream genes, and significantly enhances iPSC generation [41]. Moreover, the miR-17-92, miR-106b-25, and miR-106a-363 clusters are highly expressed in the early phases of somatic cell reprogramming and directly target PTEN, p21, and TGFβR2, resulting in promoted iPSC induction by accelerating MET, cell cycle transitions, and regulation of epigenetic factors [11, 42]. In the reprogramming of somatic cells, miRNAs are more likely to act as co-factors by enhancing the reprogramming process, as shown with miR-524-5p in this work, rather than acting in solo to exert their effects. It also seems likely that different miRNA-driven regulatory mechanisms and pathways may be involved in reprogramming normal somatic cells as opposed to cancer cells, as in the case of miR-520d-5p and hepatoma [15].

Furthermore, we also observed in this study the lack of inverse correlation in expression levels between miR-524-5p and TP53INP1 in some cell lines, suggesting that besides regulation by miR-524-5p, TP53INP1 expression is likely regulated by other factors, notably by different transcription factors. Upon exposure to genotoxic agents, TP53INP1 expression was previously shown to be induced through p53-dependent and p53-independent pathways by transcription factors p53 and p73, respectively [32, 43, 44]. Furthermore, the transcription factor E2F1 directly upregulates TP53INP1 transcription independent of p53 and p73 [44], as do the inflammatory mediators tumor necrosis factor alpha and interleukin-6 [45]. On the other hand, recent studies have also demonstrated that TP53INP1 expression is often downregulated or silenced in cancer cells by numerous onco-miRNAs including miR-130b, miR-155, and miR-125b which are present in tumor cells [35, 46, 47]. In short, TP53INP1 is subjected to both positive and negative regulation by a battery of transcription factors and miRNAs, including miR-524-5p as shown in this work.

TP53INP1 and p53 are involved in many cellular processes, including apoptosis and regulation of cell cycle and ROS-induced stress [32, 43]. On induction by p53, TP53INP1 is SUMOylated and, in turn, regulates p53 transcriptional activity by targeting antiproliferative and proapoptotic genes, such as p21, Bax, and PUMA, leading to cell-cycle arrest at the G1 phase or apoptotic cell death [32]. In another study, TP53INP1 was also shown to regulate p53 activity on genes related to cell-cycle regulation (Mdm2 and p21) and apoptosis (Pig3 and Bax) [48]. Hence, TP53INP1 and p53 form a positive feedback loop in their action. Furthermore, ectopic expression of miR-504, miR-33, and miR-1285 has been shown to induce phenotypic changes associated with the loss of p53, including reduced apoptosis and increased stemness [49]. Data from this and other works therefore strongly indicate that miRNA modulating the expression of both the TP53INP1 and p53 genes is important in fine-tuning the regulation of cell proliferation and apoptosis in the induction of pluripotency in iPSCs.

miR-524-5p was also shown in this work to upregulate the expression of pluripotency genes Oct4, Nanog, Sox2, and Rex1 (Fig. 5f). Expression of Oct4, Nanog, and Sox2 is known to be negatively regulated by p53 [1, 50, 51] and Rex1 expression is, in turn, regulated by Nanog, Oct4, and Sox2 [52, 53]. Hence, it may be speculated that the miR-524-5p/TP53INP1-induced upregulated expression of pluripotency genes observed in this study may be a consequence of TP53INP1-induced p53 repression.

To achieve successful iPSC induction, exogenous factors are needed to initiate the MET program at the early stage of process by inhibiting EMT signals and activating the epithelial program [3, 54]. In this study, miR-524-5p was found to promote MET by inhibiting the expression of the EMT-related genes SMAD4 and ZEB2 (Fig. 6), which may thereby be associated with enhancing the reprogramming process. More specifically, reprogramming has been reported to be associated with the loss of the somatic cell signatures, such as expression of the transcription factors SNAIL1/2 or ZEB1/2, and the gain of epithelial signatures, including expression of E-cadherin [3]. A SNAIL1-SMAD3/4 complex has previously been shown to promote the TGFβ-mediated downregulation of E-cadherin while ZEB2 regulates repression by binding to the E-box motif of the regulatory sequence of the E-cadherin gene [54]. Similarly, miR-302/367 and miR-200 play a crucial role in iPSC generation by targeting EMT-related genes TGFβR2 and ZEB1/ZEB2, respectively [12, 14], echoing our finding of miR-524-5p regulation of ZEB2.

**Fig. 7:**
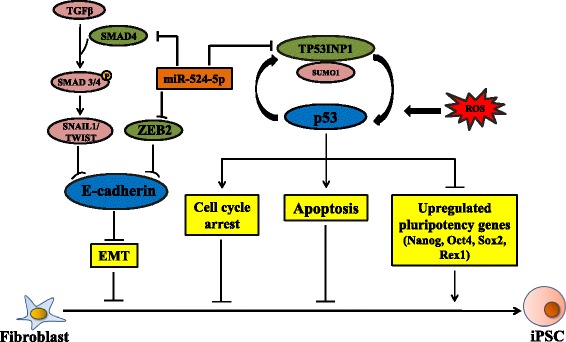
A proposed scheme of miR-524-5p regulation of the early stage of the reprogramming process [2, 3]. In the scheme, miR-524-5p promotes reprogramming by downregulating TP53INP1 to mediate processes associated with cell cycle, apoptosis, and expression of pluripotency genes, which are essential for the early stage of reprogramming. Furthermore, miR-524-5p also enhances MET, a required process for initial reprogramming, by targeting the EMT-related genes ZEB2 and SMAD4. See text for further description of the proposed scheme. *EMT* epithelial-to-mesenchymal transition, *iPSC* induced pluripotent stem cell, *ROS* reactive oxygen species

Taken together, a scheme is proposed here to summarize the involvement of miR-524-5p in the reprogramming process via interactions with TP53INP1, ZEB2, and SMAD4, and the subsequent regulation of the p53 circuitry (Fig. 7). In this scheme, miR-524-5p suppresses SMAD4 and ZEB2 resulting in upregulation of the MET marker E-cadherin via the TGFβ pathway or by direct suppression of E-cadherin, respectively. On the other hand, direct suppression of TP53INP1 expression by miR-524-5p also leads to the p53 ablation, which in turn causes downregulation of a cascade of p53-targeted genes involved in the cell cycle arrest and apoptosis, but upregulates expression of pluripotency genes. Included in the scheme is the previously reported ROS-induced p53 activation to form a feedback loop in the activation of TP53INP1 [32, 37].

## Conclusions

In this work, we have provided experimental evidence to support that the C19MC miR-524-5p targets TP53INP1 to enhance cell proliferation and to suppress apoptosis, which are critical events in the early phase of cellular reprogramming. Our data also show that miR-524-5p targets the EMT-associated SMAD4 and ZEB2 genes to suppress MET, which is also a crucial step in initiating reprogramming. Other C19MC miRNAs, particularly those that share the same seed sequence with the known reprogramming miR-302/-372 families [7], may also be shown to contribute to cellular reprogramming in future studies.

## Additional files

Additional file 1: Table S1. mRNA primers. (DOCX 19 kb)

Additional file 2: Table S2. Primers for cloning of luciferase constructs. (DOCX 21 kb)

## Abbreviations

AP: Alkaline phosphatase
BrdU: 5-Bromo-2’-deoxyuridine
C19MC: Chromosome 19 miRNA cluster
DMEM: Dulbecco’s modified Eagle’s medium
Dox: Doxycycline
ELISA: Enzyme-linked immunosorbent assay
EMT: Epithelial-to-mesenchymal transition
ESC: Embryonic stem cell
FBS: Fetal bovine serum
iPSC: Induced pluripotent stem cell
KEGG: Kyoto Encyclopedia of Genes and Genomes
KOSR: Knockout serum replacement
MEF: Mouse embryonic fibroblast
MET: Mesenchymal-to-epithelial transition
miRNA: microRNA
MSC: Mesenchymal stem cell
MTT: 3-(4,5-Dimethylthiazol-2-yl)-2,5-diphenyltetrazolium bromide
NC: Negative control
NEAA: nonessential amino acids
nt: Nucleotide
OSKM: Oct4, Sox2, Klf4, and c-Myc
PCR: Polymerase chain reaction
ROS: Reactive oxygen species
siRNA: Small interfering RNA
UTR: Untranslated region
WJ0706: Wharton’s Jelly MSC 0706
