# Supplementary material for: miR-524-5p of the primate-specific C19MC miRNA cluster targets TP53IPN1- and EMT-associated genes to regulate cellular reprogramming
Source: Stem Cell Res Ther. 2017 Sep 29;8:214. doi: 10.1186/s13287-017-0666-3 (PMC5622517; doi:10.1186/s13287-017-0666-3)
Supplement: Supplementary file 1 — mRNA primers. (DOCX 19 kb) [file 13287_2017_666_MOESM1_ESM.docx]

**Additional file 1: Table S1: mRNA primers (5’-3’)**

| TP53INP1 | F: GCC CCA CGT ACA ATG ACT CTT CT  R: GCC CTT CTT GGT TGG AGG AAG AAC |
| --- | --- |
| ZEB2 | F: ATATGGTGACACACAAGCCAGGGA  R: GTTTCTTGCAGTTTGGGCACTCGT |
| SMAD4 | F: ACTTGGCATCTCTACATTGTCC  R: GCCACATCTATTTTGCTTGCT |
| p53 | F: AGC TGA ATG AGG CCT TGG AAC T  R: AGG CCC TTC TGT CTT GAA CAT |
| Oct4 | F: GAC AGG GGG AGG GGA GGA GCT AGG  R: CTT CCC TCC AAC CAG TTG CCC CAA AC |
| Sox2 | F: GGG AAA TGG GAG GGG TGC AAA AGA GG  R: TTG CGT GAG TGT GGA TGG GAT TGG TG |
| Nanog | F: AGT CCC AAA GGC AAA CAA CCC ACT TC  R: TGC TGG AGG CTG AGG TAT TTC TGT CTC |
| Rex1 | F: TCT GAG TAC ATG ACA GGC AAG AA  R: TCT GAT AGG TCA ATG CCA GGT |
| GAPDH | F: GAA ATC CCA TCA CCA TCT TCC AGG  R: GAG CCC CAG CCT TCT CCA TG |
