# Supplementary material for: miR-524-5p of the primate-specific C19MC miRNA cluster targets TP53IPN1- and EMT-associated genes to regulate cellular reprogramming
Source: Stem Cell Res Ther. 2017 Sep 29;8:214. doi: 10.1186/s13287-017-0666-3 (PMC5622517; doi:10.1186/s13287-017-0666-3)
Supplement: Supplementary file 2 — Primers for cloning of luciferase constructs. (DOCX 21 kb) [file 13287_2017_666_MOESM2_ESM.docx]

**Additional file 2: Table S2**: Primers for cloning of luciferase constructs

| **Primer designation** | **Sequence (5’ > 3’)** |
| --- | --- |
| 1. **Primers for wild-type constructs** | |
| TP53INP1-F1 | TAA GCA **GAG** **CTC** CAG TGT TTG GGG GTG TCT TT |
| TP53INP1-R1 | CCG TGG **TCT** **AGA** AAT TGG CGG GAA GGA ATA GT |
| TP53INP1-F2 | TAA GCA **GAG CTC** ACA CGG CGT CTC TTT TTC AT |
| TP53INP1-R2 | CCG TGG **TCT AGA** AAT GCA TTT TGG CCA TGT TT |
| TP53INP1-F3&4 | TAA GCA **GAG CTC** GGG AGG TTA GAT GTG TGT TT |
| TP53INP1-R3&4 | CCG TAC **TCT AGA** GTA ACT CCA GGT AGT GCA AA |
| ZEB2-F | TAA GCA **GAG CTC** GCG GTT CAG CCA AGA CAG AT |
| ZEB2-R | CCG TGG **TCT AGA** ACT GAA GCT GGT GCA AAG GT |
| SMAD4-F | TAA GCA **GAG CTC** TCT CTT TGG AGC CAA GCC AC |
| SMAD4-R | CCT TGG **TCT AGA** GGC CTA GGA TGC CAC TTT GT |
| 1. **Primers for mutant constructs** | |
| INP1-mut1-24-F | TTCATTTTCATTTTATgaatTcTTACTTAATCTTTTAAGCAAGCA |
| INP1-mut1-24-R | TGCTTGCTTAAAAGATTAAGTAAgAattcATAAAATGAAAATGAA |
| INP1-mut2-24-F | GCCTTACCTGGGGCTAGTTTTTTATGCgaatTcCCTAGAAAAC |
| INP1-mut2-24-R | GTTTTCTAGGgaAttcGCATAAAAAACTAGCCCCAGGTAAGGC |
| INP1-mut3-24-F | CTGATTGGTTCATAGATGGTCAGTgaatTcCACAGACTGAAC |
| INP1-mut3-24-R | GTTCAGTCTGTGgAattcACTGACCATCTATGAACCAATCAG |
| INP1-mut4-24-F | TGTGTGTTAACACCTGTTCgaatTcATTGGGTTGTGGTGCAT |
| INP1-mut4-24-R | ATGCACCACAACCCAATgAattcGAACAGGTGTTAACACACA |

In the primers for the wild-type constructs, bold letters indicate the embedded cloning sites; in the mutant set, lower-case letters indicate the mutated sequences, which conveniently generated an EcoRI cleavage site.
